# Fentanyl-induced cortical and cardiopulmonary damage linked to immune response functions and apoptosis-necrosis networks in a multi-omics mouse model

**DOI:** 10.3389/fimmu.2026.1694651

**Published:** 2026-03-23

**Authors:** Nabarun Chakraborty, Mital Patel, Swapna Kannan, Candace Moyler, George Dimitrov, Aarti Gautam, Mackenzie Newman, Tara B. Hendry-Hofer, Jonathan Boyd, Rasha Hammamieh

**Affiliations:** 1 Medical Readiness Systems Biology, Center for Military Psychiatry and Neuroscience (CMPN), Walter Reed Army Institute of Research, Silver Spring, MD, United States; 2 Oak Ridge Institute for Science and Education (ORISE), Oak Ridge, TN, United States; 3 Culmen International, Alexandria, VA, United States; 4 GDIT, Culmen International, Alexandria, VA, United States; 5 Office of the Vice President for Research and Innovation, Virginia Commonwealth University School of Medicine, Richmond, VA, United States; 6 Department of Emergency Medicine, Center for Combat Research, University of Colorado School of Medicine, Aurora, CO, United States; 7 Department of Pharmaceutical Sciences, University of Colorado Skaggs School of Pharmacy, Aurora, CO, United States

**Keywords:** functional analyses, mouse model, multi-omics, opioid, system biology, fentanyl

## Abstract

**Introduction:**

Fentanyl can rapidly impair brain and cardiopulmonary functions due to its high pharmacokinetics, necessitating a systems-level investigation to elucidate the early host response profile. To address this, we developed an SKH-1 mouse model to integrate *ex-vivo* imaging with multi-omics data, enabling a comprehensive understanding of tissue-specific host responses across time and dose gradients.

**Methods:**

Our previous study characterized the phenotypes of this mouse model to establish dose gradients and time points associated with major clinical manifestations. Building on these findings, cortex, heart, and lung tissues were collected postmortem at 40 min, 6h, 24h, and 7 days following administration of one of three fentanyl doses: the highest non-lethal dose (HNLD), LD10, and LD50.

**Results:**

Multi-omics analysis revealed immune response networks and apoptosis-necrosis functions as primary targets of fentanyl. Cortical and pulmonary immune responses exhibited dose-dependent latencies but remained activated 7 days post-exposure, whereas the cardiac immune response was suppressed over time. Pulmonary apoptosis-necrosis was rapidly activated, contrasting with its delayed, dose-dependent activation in the heart. In the cortex, apoptosis-necrosis followed a monophasic longitudinal trajectory, with delayed activation after 24h followed by regression. These findings suggest tissue-specific time windows for early intervention. Subsequent machine learning analysis identified phylogenetically conserved and miRNAs, such as miR-146-5p and miR-877-3p, which demonstrated consistent time- and dose-independent regulation in the lungs and cortex, respectively.

**Conclusion:**

Functional associations of these miRNAs with tissue-specific lesions highlight their potential therapeutic value. Further interrogation of miRNA-mRNA interactions and downstream target analysis could pave the way for developing precision countermeasures against fentanyl toxicity.

## Introduction

The opioid epidemic, fueled by the misuse of ultrapotent synthetic opioids like fentanyl, has emerged as a global public health crisis (1, 2). This crisis is further exacerbated by polydrug use, where fentanyl is co-administered with substances like nicotine, cannabis, and cocaine, significantly increasing toxicity and mortality risk (3). Fentanyl’s pharmacokinetics, characterized by a short distribution half-life (~3 min) (4), enables rapid cardiopulmonary impairment and blood-brain barrier penetration. This swift central nervous system penetration triggers severe clinical manifestations, including opioid-induced respiratory depression (OIRD), muscle rigidity, and wooden chest syndrome (WCS) (5, 6)—a life-threatening condition unique to fentanyl. Even a sub-lethal dose of fentanyl can trigger a rapid hypoxia that gradually shifts to the hyperoxic phase (7). Fentanyl’s higher lipophilicity (8) enables it to rapidly bind to both target and off-target receptors (9) that significantly narrows the therapeutic window for intervention, thereby limiting the efficacy of naloxone (10), an FDA-approved opioid countermeasure.

Emerging research highlights the need for integrated models combining pharmacogenomics, pharmacokinetics, and multi-omics analyses to better understand the complex pathophysiology of fentanyl toxicity (11–13). Isolated studies have identified organ-specific biomarkers in the cardiopulmonary system (14) and brain subregions associated with pain and cognitive functions (15–17), as well as tissue-specific dysregulation of cytokines, chemokines, and growth factors (18). However, a significant knowledge gap remains in understanding the coordinated response of these organs to fentanyl dose gradients.

The present study built upon our previous reports on the lethality of fentanyl in SKH-1 mice, a hairless yet immunocompetent outbred strain that is particularly suited for imaging and omics research (18, 19). These past studies reported the following: (i) fentanyl doses were determined as HNLD: 62 mg/kg, LD10: 110 mg/kg, and LD50: 135 mg/kg. (ii) The LD50 cohort exhibited decreased breaths per minute, minute volume, and inspiratory quotient compared to the LD10 cohort. (iii) The average time to death following LD50 exposure was 44.9 ± 7.4 min, introducing a potential “survivor bias,” as discussed later. (iv) Reduced glucose uptake was observed in the brain, heart, and lungs, persisting up to 24h post-exposure. (v) *Ex-vivo* imaging identified the brain cortex, heart, and lungs as early targets of fentanyl exposure (19). These observations align with the fact that WCS is a unique signature of fentanyl (5, 6) and that the cortex, a major brain region responsible for pain management and sensing, has been extensively studied in opioid-related research (20, 21). Leveraging this knowledge, the current study undertook the transcriptomics and non-coding RNA profiling, including miRNAs, to evaluate tissue-specific molecular changes and track longitudinal responses across dose gradients in these three tissues.

A growing number of studies highlights the emerging role of miRNAs as regulators of diverse physiological processes (22), including pain perception, somatosensory networks, and cardiopulmonary functions, all of which are disrupted by fentanyl overdose. Preliminary evidence links miRNAs to fentanyl’s analgesic effects (23), and the recent FDA approval of miRNA-based therapies for central nervous system and cardiovascular diseases (24) underscores their potential as countermeasures for opioid toxicity. Despite these promising leads, translating preclinical findings to clinical applications remains a major challenge, with more than 85% of animal data failing to achieve clinical success (25). To address this, our group has developed a machine learning-enabled pipeline to identify molecular features with sequential and functional homology across species (26). This approach, previously validated in radiation research (27, 28), was applied to fentanyl toxicity to enhance translational relevance.

In summary, this study offers a comprehensive analysis of tissue-specific molecular responses to fentanyl overdose, emphasizing the role of miRNAs as key regulators and therapeutic candidates. By bridging the gap between preclinical research and clinical application, the findings pave the way for targeted interventions to mitigate fentanyl toxicity and address the growing opioid epidemic.

## Materials and methods

### Animal model established by past work

**Figure 1:**
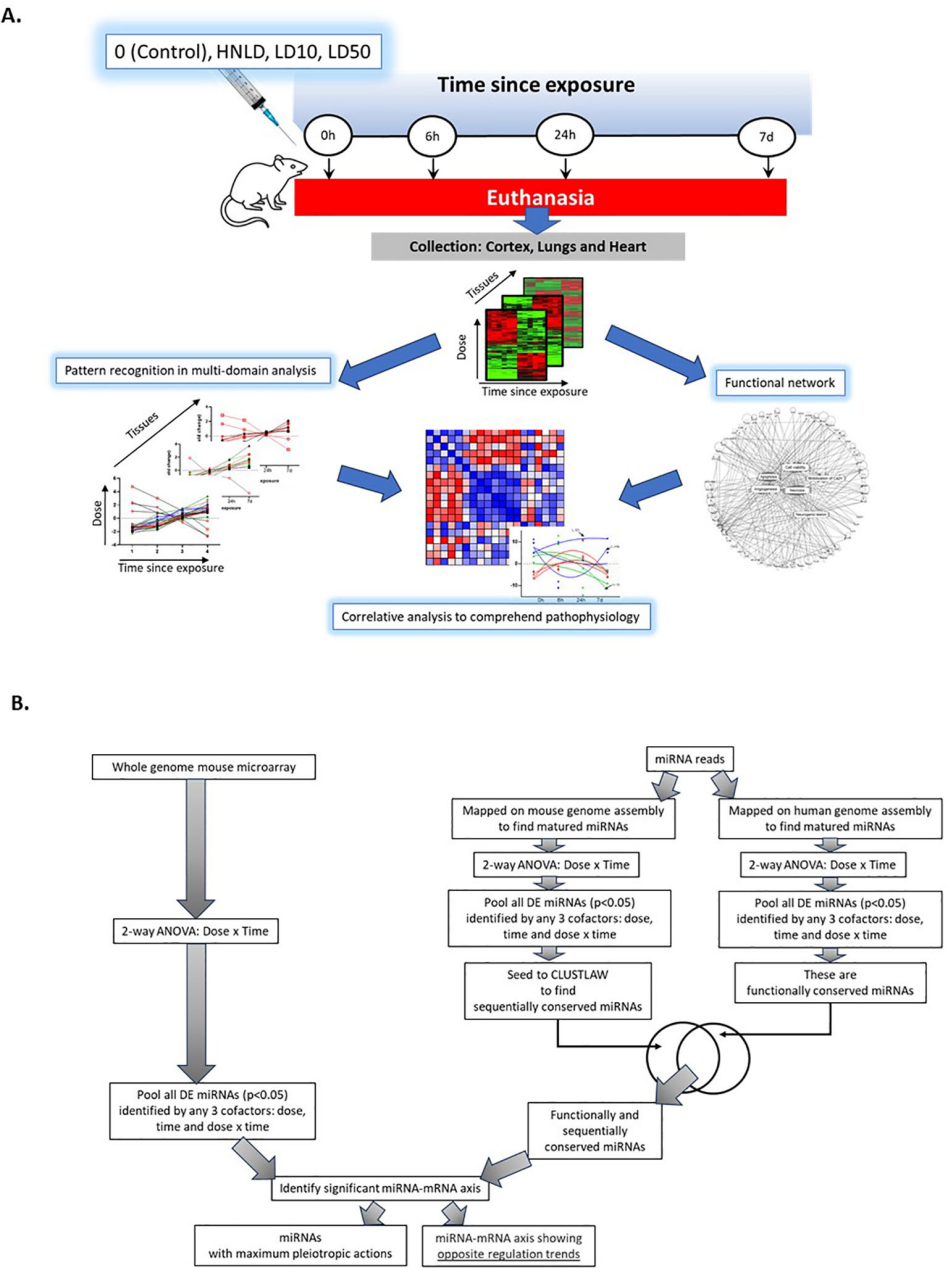
Overall study design. **(A)** Pictorial depiction of the study protocol. **(B)** Flowchart of the analytical pipeline leading towards integrating mRNA and miRNA data. The left arm showed the revised pipeline (26) to screen miRNA data to find features of high translational potential.

The outline of this study design is presented in **Figure 1A**, and the detailed procedure has been reported elsewhere (18, 19). Adult SKH1 Elite mice (strain code 477, Charles River, Wilmington, MA) were group-housed in standard vivarium conditions with food and liquid *ad libitum*. Fentanyl citrate in Ringer’s solution was administered via subcutaneous injection with an increasing dose that included (i) the highest non-lethal dose (HNLD): 62 mg/kg; (ii) LD10: 110 mg/kg and (iii) LD50: 135 mg/kg. Each group had four mice, and sham-control mice received vehicles only. The method and corresponding results to test lethality, to monitor pulse oximetry (SpO_2_ and heart rate), and the whole-body plethysmography have been reported earlier (19).

### Current animal model following past study template

An independent set of mice (*n* = 4/group) underwent the same protocol and were humanely euthanized at 40 min, 6h, 24h, or 7 days post-exposure. Henceforth, the euthanasia that took place 40 min post-exposure is designated as “0h.” The post-mortem organs, including the brain cortex, heart, and lungs, were extracted at 0h, 6h, 24h, and 7 days post-administration. The baseline sham cohort was euthanized at the same timepoints. The tissues were snap-frozen at −80 °C until they were used for omics assays. To note, this project processed 64 mice altogether.

All animal procedures were conducted in compliance with the U.K. Animals (Scientific Procedures) Act, 1986, EU Directive 2010/63/EU, and the National Research Council’s *Guide for the Care and Use of Laboratory Animals*. Experiments were reported in accordance with the ARRIVE guidelines. Research was conducted under protocols approved by the West Virginia University and Virginia Commonwealth University Institutional Animal Care and Use Committee (IACUC) and the U.S. Army Animal Care and Use Review Office (ACURO). All work was performed in AAALAC International-accredited facilities with Public Health Service Animal Welfare Assurance and in full compliance with the Animal Welfare Act and all applicable federal regulations.

### Gene transcriptomic assay

This assay was performed following our established protocol (29). TRIzol reagent (Invitrogen, Thermo Fisher Scientific, Wilmington, MA) was added to the tissues, and the specimens were homogenized using a Precellys Evolution Homogenizer. The Invitrogen TRIzol RNA extraction method and the miRNeasy Mini Kit total RNA purification procedure (QIAGEN Inc., Germantown, MD) were followed to extract the miRNA and total RNA. The quantity and quality of RNA were then determined using a NanoDrop 2000 spectrophotometer (Thermo Fisher Scientific, Wilmington, DE), Qubit 3.0 fluorometer, and 2200 TapeStation System (Agilent Technologies, Inc., Santa Clara, CA), respectively. The purified RNA samples were stored at −80 °C for future use.

Dual-dye microarrays were performed using the SurePrint G3 Mouse Gene Expression 8 × 60 k Microarray Kit (Agilent Technologies, Inc., CA, USA). This whole mouse genome microarray slide contains 41,000 mouse transcripts. Briefly, purified RNA was labeled with Cy-5 dyes. The mouse reference RNA (Agilent Technologies, Inc.) was labeled with Cy-3 dyes. The samples were hybridized and incubated for 17h at 65°C. After overnight incubation, the slides underwent a series of washes. The slides were imaged using an Agilent SureScan Microarray Scanner, and the features were extracted using the default settings of the manufacturer software (Feature Extraction software v.10.7, Agilent Technologies, Inc.).

### Gene transcriptomic data analysis

The overall strategy for data analysis is shown in **Figure 1B**. Three tissues were analyzed independently. Unsupervised Principal Component Analysis (PCA) was measured, and two-way repetitive analysis of variance (ANOVA) followed by post-Tukey was calculated for the dependent variables, namely the fentanyl dose, the time since exposure, and their cumulative effects, dose × time; the significance level was set at *p* < 0.05. The differentially expressed genes were further organized by the k-means algorithm to select those gene-clusters that consistently changed their regulations over the time course. Subsequently, the selected gene clusters underwent the linear regression-based screening to find those features that showed significant changes (*p* < 0.05) across the time course. These selected pools of genes were further screened to exclude those that were regulated between log_2_(fold change) +1 and −1 within the entire time course from 0h to 7d post-exposure.

Those genes, which were differentially expressed by the cumulative impacts of dose and the time since exposure (dose × time), were uploaded to Ingenuity Pathway Analysis (IPA) (Qiagen, Inc.) for functional analysis. Regulation of a gene network was determined by the z-score, and the selections were undertaken based on the following two criteria: z-score ≥ |1| and the number of genes enriching the network should be more than 5. The z-scores of these screened networks were plotted against the time course, and a second-order polynomial least squares fit model was used to identify their longitudinal regulation patterns; *R*^2^ values were calculated to measure the fitting efficacies. To understand the interplay among these three tissues, namely, the cortex, heart, and lungs, a correlative analysis was performed at the gene functional level. The Pearson correlation matrix was screened based on r > |0.5| and *p* < 0.05.

### microRNA profiling

From the aliquot of total RNA, miRNA profiling was conducted following our established protocol (30). Two hundred and fifty nanograms of total RNA were used to construct sequencing libraries using an Illumina (Illumina Inc., CA, USA) TruSeq Small RNA Library Prep Kit, following the manufacturer’s guidelines. Briefly, 3′ and 5′ adapters were sequentially ligated to small RNA molecules, and the ligated products were reverse transcribed, amplified, pooled, and subsequently size-selected by gel purification. The library pools were quantified using the KAPA Library Quantification Kit for Illumina Platforms (Roche Diagnostics Corporation, Indianapolis, IN), diluted, and sequenced on the Illumina NextSeq 500 Sequencer to generate 50 base-pair reads. Image analysis and base calling were performed using the Illumina pipeline Version 1.5.15.1 and Illumina CASAVA sequencing analysis software Version 1.7.32.0. The raw sequence reads were processed using a robust miRNA-seq quality control pipeline. First, the samples were evaluated as per quality control assurance using matrices that included acceptable duplication, k-mer, or GC content generated using FASTQC. The samples that showed more than 30% disagreement with the rest of the samples were excluded. The low-quality reads (N20 quality score threshold) were filtered out, and adapter sequences were pruned. The ensuing products were 48%–75% mapped against the mature miRBase miRNA database version 21 for Mus musculus (mm9). A pool of RNA species was curated, and the effective library sizes were normalized using the trimmed mean of M-values (TMM) normalization method provided by edgeR (Bioconductor.org). In the second phase of filtration, the samples with less than 1% abundance were also discarded. Meanwhile, we validated the miRBase output by mapping the same filtered sequence reads against the UCSC reference genome for Mus musculus (mm9 build) (University of California, Santa Cruz; http://genome.ucsc.edu/). The short read aligner Bowtie (v1.0.0) allowed one pair of mismatches.

### miRNA data analysis

Unsupervised principal component analysis (PCA) was measured using the small RNA reads. Subsequently, these reads were processed via a machine learning algorithm (26) to find features with high translational potential. Described in **Figure 1B**, one arm of this pipeline identified *sequentially conserved* miRNAs between human and mouse. Towards this purpose, a two-way ANOVA was performed on the miRNA reads to determine miRNAs significant with dose × time (*p* < 0.05). This subset was then analyzed using ClustalW in the msa (31) R package to find the best human miRNA sequence match for each mouse miRNA. The following parameters were used while running ClustalW: type=“RNA,” ktuple= 1, pairgap= 10, window=5, topdiags=5, and score=“PERCENT.” Additionally, the log2fold change was determined using the edgeR package. The second arm in **Figure 1B** curated *functionally conserved* miRNAs, which would have been similarly perturbed in both humans and mice under fentanyl exposure. Here, the reads were mapped on the human genome, as the reference was obtained from miRBase, version 22.1. The human miRNA from the mature.fa files was used. Subsequently, a two-way ANOVA was computed to find miRNAs that were affected by dose × time (*p* < 0.05).

A Venn diagram compared these two sets of miRNAs, namely, the *sequentially conserved* and *functionally conserved* miRNAs, to curate the overlapping features, which would demonstrate sequential homology between these two species’ molecular makeups and functional similarity in responding to the stress. The *conserved* miRNAs were identified for the three tissues, cortex, lungs, and heart, and further analysis only used these *conserved* miRNAs. Downstream targets of the *conserved* miRNAs were identified by mining four databases, namely TargetScan, TarBase, miRecords, and the Ingenuity Knowledge Base, to find those mRNAs that were typically targeted by the *conserved* miRNAs. The list of miRNA–mRNA duplexes was further screened to identify those miRNA:mRNA pairs, that showed opposite regulations (30).

The mRNA and miRNA databases are available in the GEO domain: GSE296051 and GSE296604, respectively.

## Results

### Global transcriptomic analysis suggests a time-dependent response

**Figure 2:**
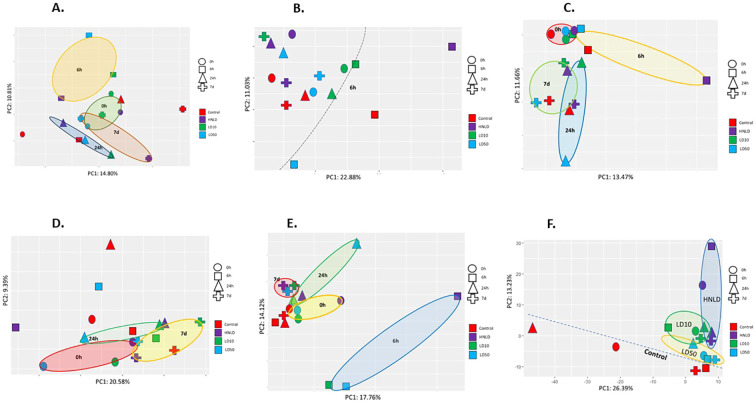
Principal component analysis (PCA). Two-dimensional PCA plots show the distribution of the samples between PC1 and PC2. The time points and doses are depicted by shapes and colors, as noted by the legend in right. Prominent clusters are highlighted. **(A)** mRNA profile of cortex. **(B)** miRNA profile of cortex. **(C)** mRNA profile of heart. **(D)** miRNA profile of heart. **(E)** mRNA profile of lungs. **(F)** miRNA profile of lungs.

Cortex: The two-dimensional PCA plot of the cortical mRNA profile explained nearly 25% of its total variance (**Figure 2A**). The fentanyl-exposed samples were primarily segregated based on the time since exposure, and the time-specific sham controls were clustered separately from their fentanyl-treated counterparts.

Heart: The two-dimensional PCA of the cardiac mRNA profile (**Figure 2C**) showed a cortex-like trend. Samples were clustered according to the time since fentanyl exposure. The samples were displayed in a two-dimensional plot that explained nearly 25% of total variance.

Lungs: The PCA of the lungs’ (**Figure 2E**) mRNA profile explained nearly 32% of total variance, and the profile was very similar to that of the mRNA PCA plots of the other two tissues.

### Tissue-specific time- and dose-dependent differential gene expression analysis

The two-way ANOVA of the cortical mRNA profile identified nine genes that were differentially expressed due to the fentanyl dose gradients; 68 genes were differentially expressed by the time since fentanyl exposure, and 249 genes were differentially expressed by the cumulative factor of fentanyl dose and time (dose × time). Likewise, the two-way ANOVA of cardiac gene expression analysis found 14 genes that were differentially expressed due to the fentanyl dose gradients; 87 genes were differentially expressed by the time since fentanyl exposure, and 175 genes were differentially expressed by the cumulative factor of fentanyl dose and time (dose × time). Finally, the two-way ANOVA of pulmonary gene expression analysis found 24 genes that were differentially expressed due to the fentanyl dose gradients, 180 genes were differentially expressed by the time since fentanyl exposure, and 301 genes were differentially expressed by the cumulative factor of fentanyl dose and time (dose × time). **Supplementary Figure 1** presents the longitudinal patterns of these differentially expressed genes. The subsequent gene regulations’ pattern search using the k-means algorithm found those genes that were significantly regulated across the time points, and the next section described this subset of genes.

Cortex (**Supplementary Figures 1A–S1C**): There were 14 genes that significantly increased and a single gene, Slc9a2 significantly decreased with time since the HNLD fentanyl administration (**Supplementary Figure 1A**). These 15 genes significantly enriched the biofunctions linked to neurogenesis of neoplasm, neuritogenesis, and G-protein-coupled receptors. Likewise, 24 genes were significantly increased, and four genes significantly decreased with time since the LD10 fentanyl administration (**Supplementary Figure 1B**). In addition, there were seven genes that significantly increased and two genes that significantly decreased with time since the LD50 administration (**Supplementary Figure 1C**). Among these genes, Slc9a2 significantly decreased with time since the exposure of both HNLD and LD10; in contrast, Tlx2 significantly increased with time since the exposure of both HNLD and LD10. Two genes, Cd1d1 and Scarb1, were significantly increased with time since the exposure of both LD10 and LD50.

Heart (**Supplementary Figures 1D, S1E**): No significantly increased or decreased genes were found across time since HNLD administration. One cardiac gene, Mob1b, significantly increased, and three cardiac genes were significantly decreased with time since LD10 administration (**Supplementary Figure 1D**). Likewise, one cardiac gene, Cggbp1, was significantly increased, and two cardiac genes significantly decreased with time since LD50 administration (**Supplementary Figure 1E**).

Lungs (**Supplementary Figures 1F–S1H**): Three pulmonary genes were significantly increased, and one pulmonary gene, namely Gpr89, was significantly decreased with time since the HNLD administration (**Supplementary Figure 1F**). In addition, five pulmonary genes were significantly increased, and one pulmonary gene, Atad2, was significantly decreased with time since the LD10 administration (**Supplementary Figure 1G**). Furthermore, seven pulmonary genes significantly increased, and three pulmonary genes significantly decreased with time since the LD50 administration (**Supplementary Figure 1H**); together these 10 genes linked to LD50 significantly enriched the biofunctions linked to neurogenesis of lung lesions and phosphorylation. Of note, two genes, namely, Gpat2 and Sox9, were significantly increased with time since both LD10 and LD50 exposure.

### Tissue-specific network analysis

There were 249 cortical genes, 175 cardiac genes, and 301 pulmonary genes, all of which were regulated by the cumulative effects of fentanyl dose and time since the fentanyl exposure (dose × time). Functional analyses were conducted using these pools of genes (**Table 1**, and detail is in **Supplementary Table 1**).

**Table 1:** Major tissue specific biofunctions.

Biofunction (# of genes)	HNLD				LD10				LD50			
	0h	6h	24h	7d	0h	6h	24h	7d	0h	6h	24h	7d
(A) Cortex												
Apoptosis/Necrosis (59)	↓	↓	↑	↓	↓	↓	↑	↓	↓	↓	⇑	↓
Pyroptosis Signal (5)	↓		↑		↓	⇓	⇑	↑	↓	↓	↑	
Oxytocin in Brain (5)			⇓				⇓			↑	↓	
Inflammatory response (11)	↓	↓	↑	↑	↓	↓	↑	↑	↓	↓	↑	↑
Proliferation of immune cells (9)	↓						↑				↑	
Quantity of Ca2+ (9)	↓		↑			↓		⇑	↓	↓		⇑
Ion homeostasis (9)	↓			↑		↓		⇑		↓		⇑
Angiogenesis (15)	↓	↑	↑	↑				⇑	↓	↓		⇑
G-Protein Coupled Receptor Signaling (12)	↓			↑		↓	↓	↑	↓			↑
(B) Heart												
Apoptosis/Necrosis (39)	↓	↑	↓	↓	↓	↑	⇓	↓	↑	↑	↓	↓
Cell survival (26)		⇑	↓		↓	⇑		↓	↓	↓		
P53 signaling (5)		⇑			↑	⇑		↓		⇑		
Proliferation of immune cells (8)	↑				↓			↓				
IL4/IL13 signaling		⇑				⇑		↑		↑	↑	
Quantity of ROS (8)				↑		↑	↑			↑	↑	
Fibrogenesis (7)	↑	↑						↓			↑	
G-Protein Coupled Receptor Signaling (5)		⇑					⇑		↓	↑		
(C) Lungs												
WNT/β-catenin Signaling (6)			↑	↓				⇓	↑			⇓
Microtubule dynamics (20)				↓	↓		↑			↓		
Cell movement (68)					⇓		↑				↑	↑
Synthesis of hormone (8)			↓	↑	↑		↑	↑	↑		↑	↑
Transport of and uptake by IGFBPs (5)			↓	⇑	↑		↓					
Fatty acid metabolism (13)	⇑		↓			⇑				↑		
Synthesis of cAMP (6)			↓	↑				↑				↑
G-Protein Coupled Receptor Signaling (13)			↓		↑		↓		↑			↓
Angiogenesis (18)			↓			↑				↑		

Each cell is color coded; blue: activated function, red: inhibited function and white: no change. ↑: z-score ≥ 1.0; ⇑: z-score ≥ 2.0; ↓: z-score ≤ −1.0; ⇓: z-score ≤ −2.0.

Cortex (**Table 1A**): Most of the cortical networks were linked to three overarching superfamilies, namely cell death, immune response, and ion regulation. By the number of genes, apoptosis-necrosis was the top-ranked network under the cell death superfamily, followed by the pyroptosis and oxytocin signaling networks. Inflammatory response and proliferation of immune cells were major networks within the immune response superfamily. Likewise, the ion regulation superfamily included the networks linked to the quantity of calcium ions and an overall ion homeostasis.

Heart (**Table 1B**): Most of the cardiac networks were linked to the two overarching superfamilies of cell death and immune response. By the number of genes, apoptosis-necrosis was the top ranked network under the cell death superfamily followed by the cell survival and p53 signaling networks. Proliferation of immune cells, IL-4 and IL-13 signals, and synthesis and quantity of reactive oxygen species were major signals within the immune response superfamily.

Lungs (**Table 1C**): Apoptosis of lung cancer was one of the most enriched networks under the cell death superfamily. Cell movement, microtubule dynamics, and metabolism of hormones were other highly enriched networks in lungs.

### Tissue-specific longitudinal trends in cell death and immune response

**Figure 3:**
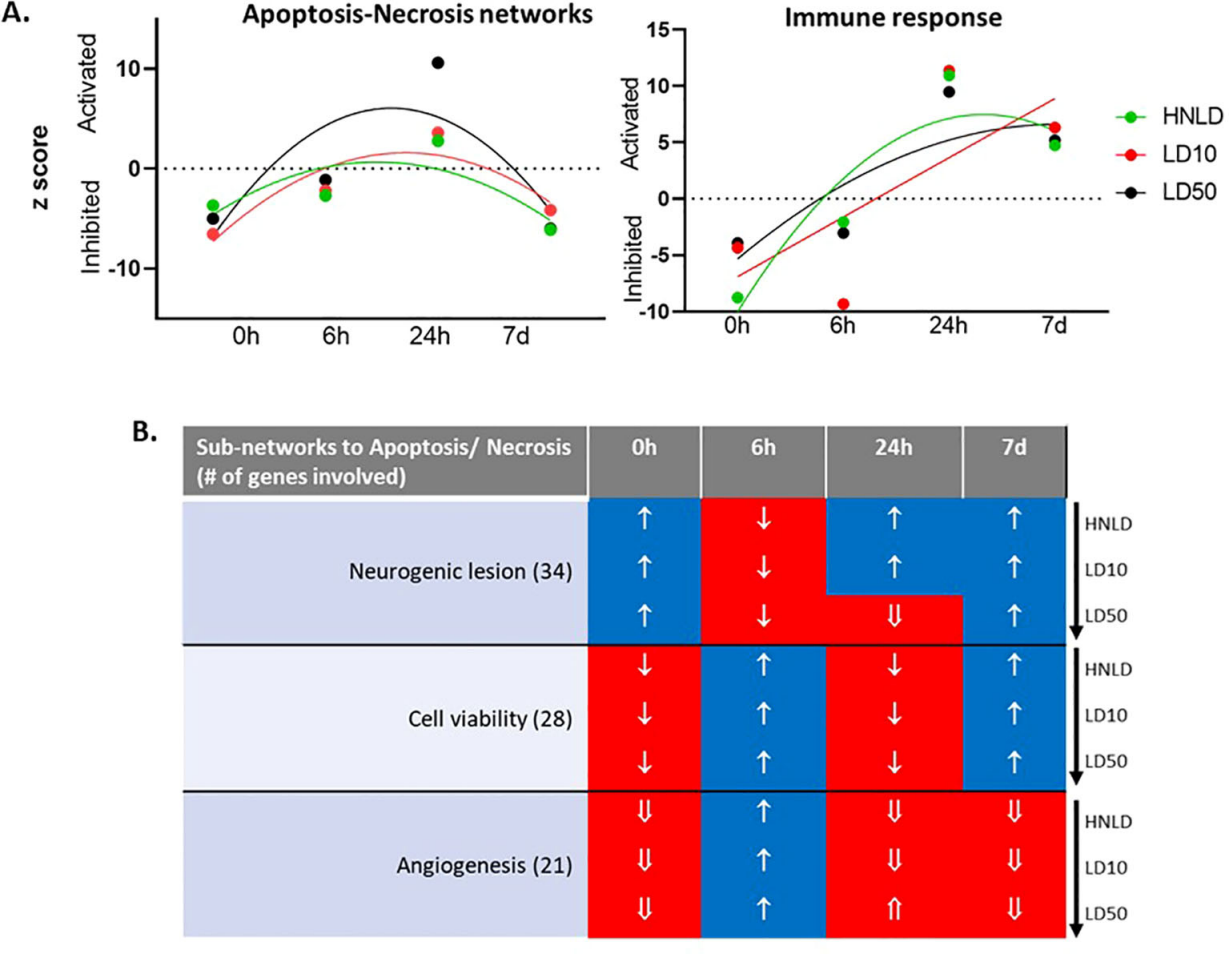
Major cortical biofunctions. **(A)** Longitudinal profile of the z-score of two networks linked to cell death and immune response. **(B)** The regulation profile of the sub-networks linked apoptosis and necrosis. ↑: z-score ≥ 1.0; ⇑: z-score ≥ 2.0; ↓: z-score ≤ −1.0; ⇓: z-score ≤ −2.0.

Cortex (**Figure 3**, **Supplementary Figure 2**): The cortical network associated with apoptosis-necrosis followed a monophasic temporal trajectory (**Figure 3A**) as computed by a second order polynomial least squares fit model (*R*^2^: HNLD = 0.58, LD10 = 0.80, LD50 = 0.62). In contrast, the network linked to immune response was linearly activated across the time (*R*^2^: HNLD = 0.85, LD10 = 0.51, LD50 = 0.68, **Figure 3A**).

The expression dynamics of apoptosis and necrosis and its major co-regulated networks, namely cell viability, neurogenic lesion, and angiogenesis, were depicted in **Figure 3B**. These 56 genes enriched a network shown in **Supplementary Figure 2A**, and the corresponding hierarchical cluster is shown in **Supplementary Figure 2D**.

**Figure 4:**
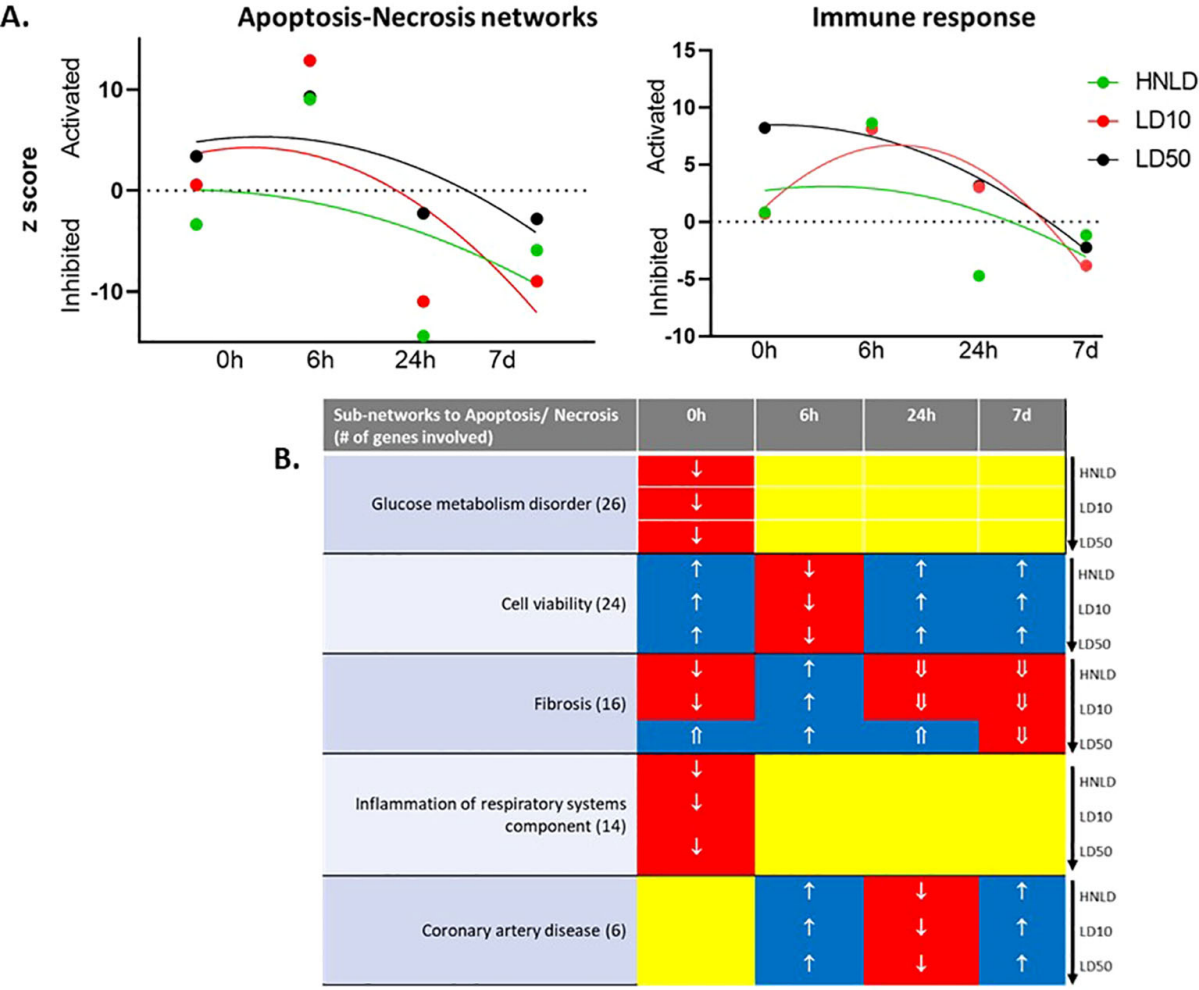
Major cardiac biofunction. **(A)** Longitudinal profile of the *z*-score of two networks linked to cell death and immune response. **(B)** The regulation profile of the sub-networks linked apoptosis and necrosis. ↑: z-score ≥ 1.0; ⇑: z-score ≥ 2.0; ↓: z-score ≤ −1.0; ⇓: z-score ≤ −2.0.

Heart (**Figure 4**, **Supplementary Figure 2**): The cardiac network associated with apoptosis-necrosis (**Figure 4A**) showed dose-dependent latencies to respond, as LD50 immediately activated this network, but HNLD inhibited it. With progression of time, this network became inhibited across all doses (apoptosis *R*^2^: HNLD = 0.18, LD10 = 0.46, LD50 = 0.58). Likewise, the network linked to cardiac immune response showed a dose-dependent latency to respond but became inhibited with time progression across all doses (*R*^2^: HNLD = 0.24, LD10 = 0.92, LD50 = 0.98; **Figure 4A**).

The expression dynamics of apoptosis and necrosis and its major co-regulated networks, namely, coronary artery disease, glucose metabolism disorder, cell viability, fibrosis, and inflammation of respiratory systems components, were depicted in **Figure 4B**. These 38 genes enriched a network shown in **Supplementary Figure 2B** and the corresponding hierarchical cluster is shown in **Supplementary Figure 2E**.

**Figure 5:**
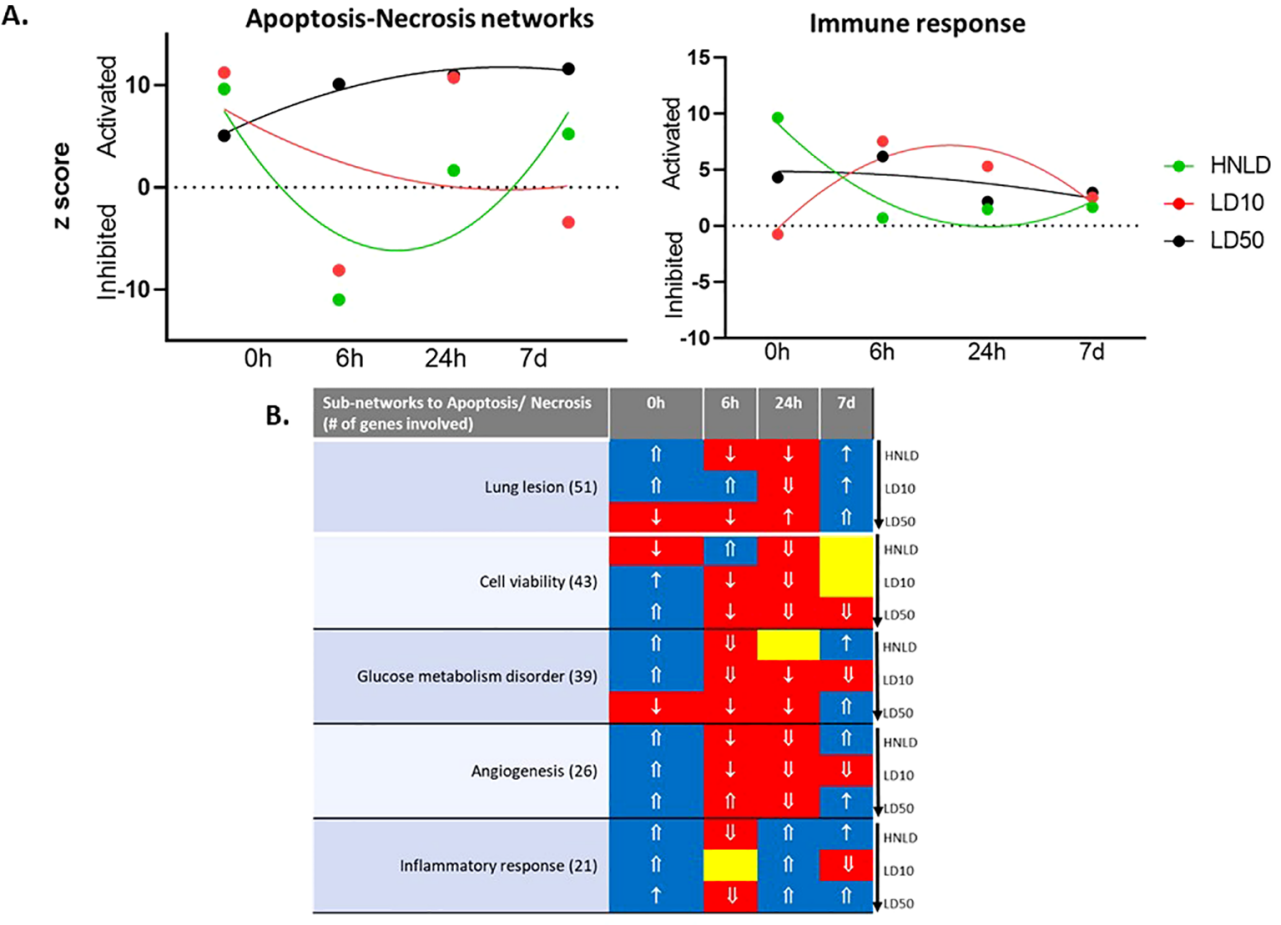
Major pulmonary biofunction. **(A)** Longitudinal profile of the z-scores of two networks linked to cell death and immune response. **(B)** The regulation profile of the sub-networks linked to apoptosis and necrosis. ↑: z-score ≥ 1.0; ⇑: z-score ≥ 2.0; ↓: z-score ≤ −1.0; ⇓: z-score ≤ −2.0.

Lungs (**Figure 5**, **Supplementary Figure 2**): The pulmonary network associated with apoptosis-necrosis (**Figure 5A**) displayed a dose-dependent trend. For HNLD, apoptosis showed a monophasic pattern, namely, its early activation was followed by inhibition and delayed re-activation; LD10 showed gradual inhibition; and LD50 showed sustained activation (apoptosis *R*^2^: HNLD = 0.62, LD10 = 0.13, LD50 = 0.97). Likewise, the pulmonary immune response dynamics showed a dose-dependent pattern. A long latency period by LD10 was followed by linear activation, while HNLD and LD50 caused persistent activation over time (immune *R*^2^: HNLD = 0.90, LD10 = 0.87, LD50 = 0.38, **Figure 5A**).

The expression dynamics of apoptosis and necrosis, and its major co-regulated networks, namely cell viability, angiogenesis, lung lesion, inflammation, and glucose metabolism disorder were depicted in **Figure 5B**. These 70 genes enriched a network shown in **Supplementary Figure 2C** and the corresponding hierarchical cluster is shown in **Supplementary Figure 2F**.

### Cross-tissue functional dynamics

**Figure 6:**
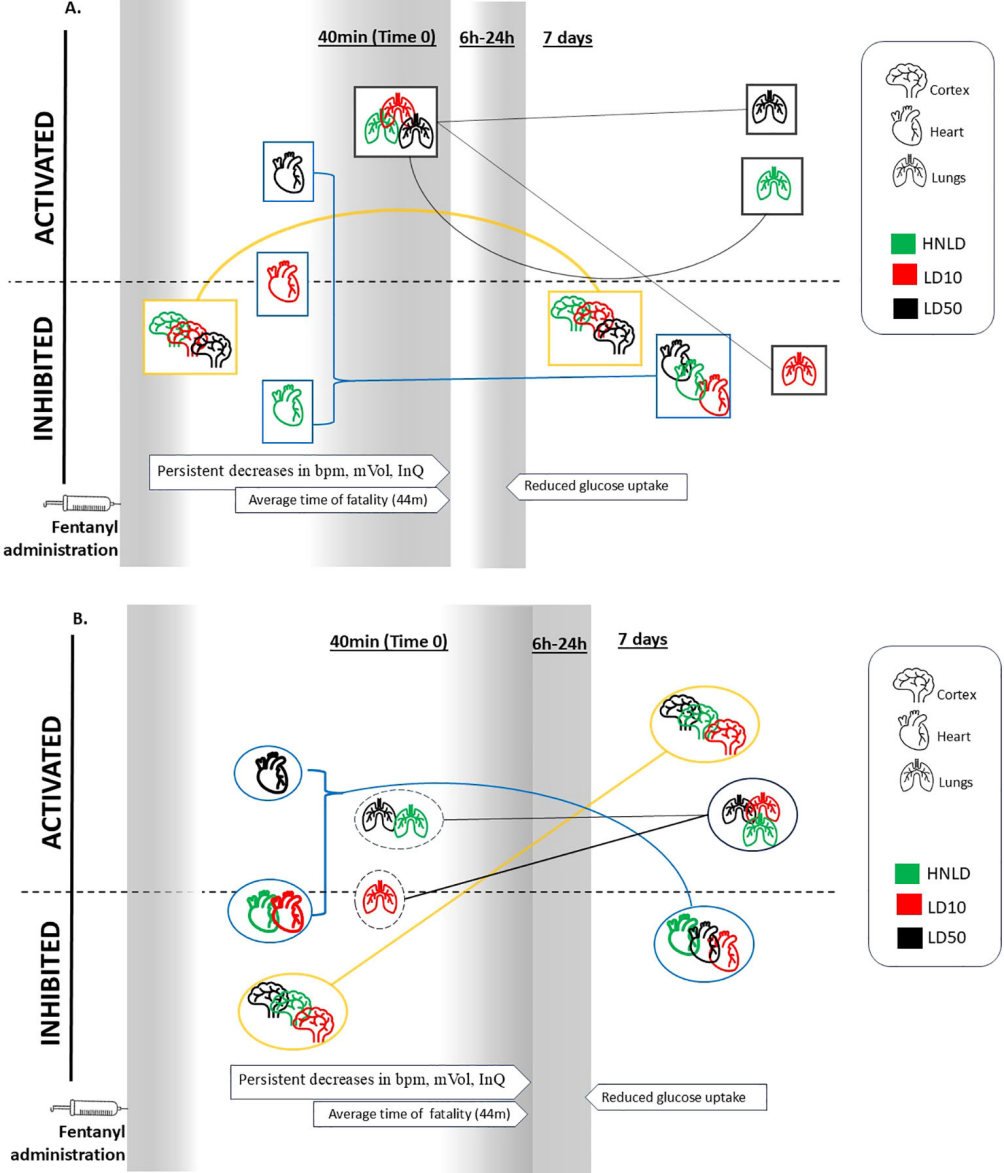
An overarching view of the tissue-specific dynamics of two networks, namely cell death and immune response. The initial (Time 0, which was 40 min after fentanyl exposure) and final (day 7 post-fentanyl exposure) time points of this study are highlighted, and the connecting lines proximately tracks the longitudinal courses of these networks. At the bottom of the figures three arrows point to certain timepoints which were associated with the events of the endpoint of persistent decreases in bpm, minute volume, inspiratory quotient, the average time of fatality due to overdose, and the time of lowest glucose uptake. **(A)** Cell death and **(B)** Immune response. bpm, breaths per minute; minute volume (mVol), number of respiratory cycles per minute; inspiratory quotient (InQ), the ratio of carbon dioxide production over oxygen consumption.

Longitudinal patterns of cross-tissue correlated networks are illustrated in **Supplementary Figure 4**. **Figure 6** presents the overall dynamics of the apoptosis-necrosis network (**Figure 6A**) and the immune network (**Figure 6B**) across varying dose and time gradients.

The longitudinal pattern of the cortical apoptosis-necrosis network linked to HNLD was positively correlated with cortical immune response (*r* = 0.83, *p* < 0.05) but negatively correlated with cardiac apoptosis-necrosis (*r* = −0.95, p < 0.05) and immune response (*r* = −0.91, *p* < 0.05), respectively. At the highest dose, LD50, the cortical apoptosis-necrosis dynamics significantly diverged from those of cortical immune response (*r* = −0.82, *p* < 0.05) and pulmonary apoptosis-necrosis network dynamics (*r* = −0.98, *p* < 0.05); on the other hand, the cortical apoptosis-necrosis dynamics were positively regulated with the immune responses in the heart (*r* = 0.82, *p* < 0.05) and lungs (*r* = 0.50, *p* < 0.05), respectively (**Table 2**, **Supplementary Figure 4**).

**Table 2:** Correlation matrix.

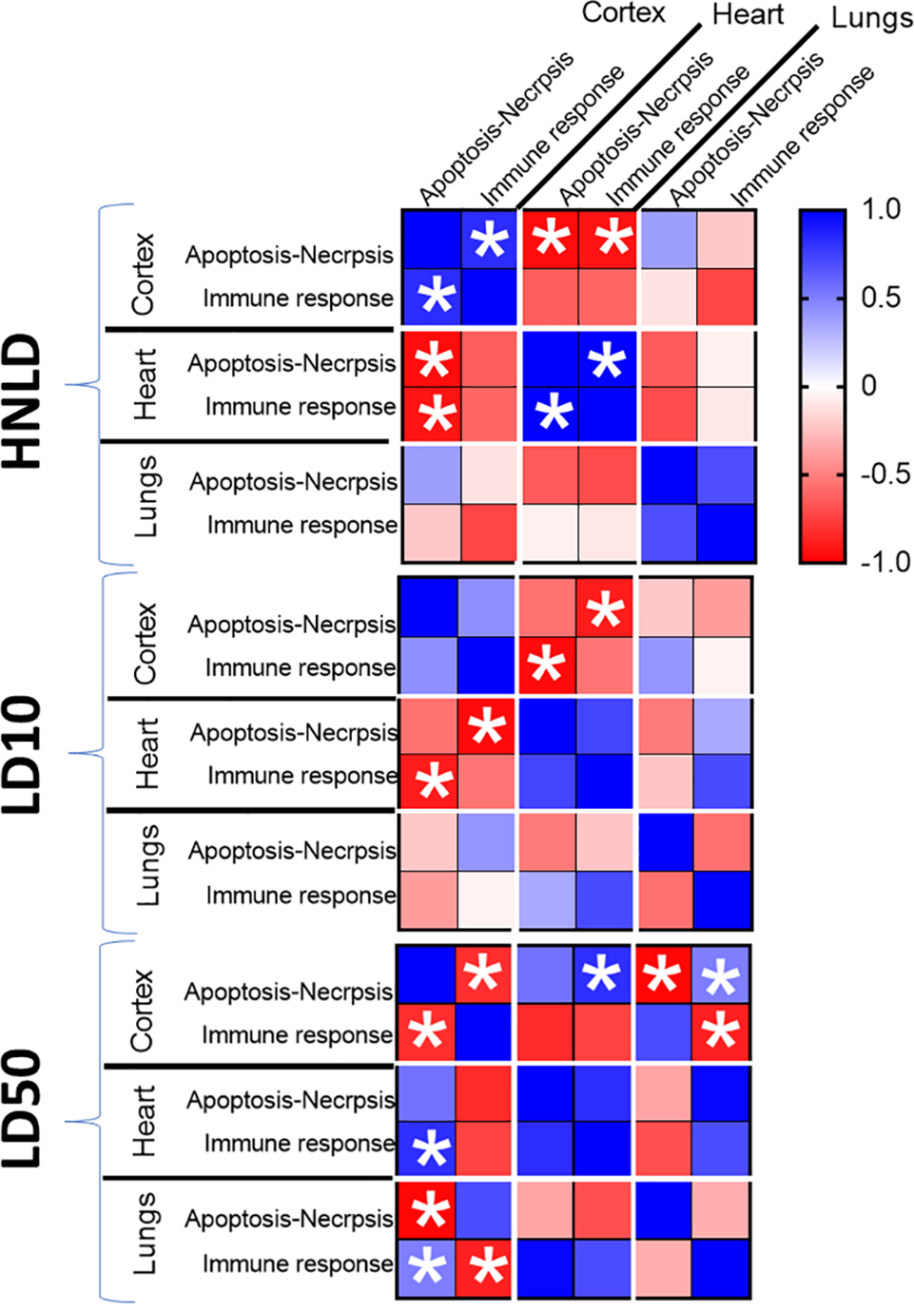

Presented values met two criteria: *r* > |0.5| and *p* < 0.05. The color bar at the right-hand side shows the distribution of r value, and the significant correlations are depicted by asterisks (*).

Major phenotypes determined by our past study (18, 19), such as the average time of fatality due to overdose and the time of lowest glucose uptake, were labeled along the network dynamics to depict their temporal alignment. For instance, at the average time of fatality due to overdose, the cell death and immune response networks were activated in the heart and lungs but inhibited in the cortex (**Figure 6**).

### miRNA profiling identifies translational candidates

The miRNA results are presented in a structured sequence, beginning with an overview of the global miRNA landscape, followed by a tissue-specific curation of differentially expressed miRNAs. Using the established machine learning algorithm (26) described in **Figure 1B**, the study identified miRNAs that are sequentially conserved and functionally similar between humans and mice. To determine sequential homology, differentially expressed rodent miRNAs were mapped onto human gene sequences, and conserved contigs were identified through pairwise alignment, as detailed in the methods section. In parallel, functional similarity was assessed by mapping the reads onto the human reference genome and computing the differentially expressed miRNAs associated with dose and time gradients. The logical integration of these two parallel analyses enabled the identification of miRNAs that are both sequentially conserved and functionally similar between humans and mice.

Finally, a miRNA–mRNA integrative analysis was performed to further explore the relationships and interactions between miRNAs and their target mRNAs.

PCA plots show time-dependent miRNA response in the cortex (**Figure 2B**) and heart (**Figure 2D**). However, the pulmonary miRNA profile emerged differently (**Figure 2F**), as these samples were clustered based on the fentanyl dose. The tissue-specific miRNA sequencing yielded 1,021 cortical reads, 648 cardiac reads, and 794 pulmonary reads.

*Cortex-specific miRNAs:* The analytical pipeline presented in **Figure 1B** identified 72 miRNAs, which were sequentially homologous and functionally similar between humans and mice (**Supplementary Table 2** and **Supplementary Figure 5A**). Of these 72 miRNAs, 6 cortical miRNAs were significantly regulated among all 12 conditions, namely, 3 doses × 4 time points (**Supplementary Figure 6**), and 2 of the miRNAs, namely miR-324-5p and miR-877-3p, were found to be the most consistently regulated miRNAs across these 12 conditions. Both miRNAs were upregulated in nine out of 12 conditions.

*Heart-specific miRNAs:* The analytical pipeline presented in **Figure 1B** identified 30 miRNAs, which were sequentially homologous and functionally similar between human and mouse (**Supplementary Table 2** and **Supplementary Figure 5B**). Further analysis found no miRNAs that were consistently regulated in the fentanyl dose and time gradient.

*Lungs-specific miRNAs:* The analytical pipeline presented in **Figure 1B** identified 135 miRNAs, which were sequentially homologous and functionally similar between human and mouse (**Supplementary Table 2** and **Supplementary Figure 5C**). Of these 135 miRNAs, 19 pulmonary miRNAs were significantly regulated among all 12 conditions, namely, 3 doses × 4 time points (**Supplementary Figure 7**). The hierarchical clustering of these 19 pulmonary miRNAs found miR-146b-5p that was consistently down-regulated across the fentanyl dose and time gradient.

**Figure 7:**
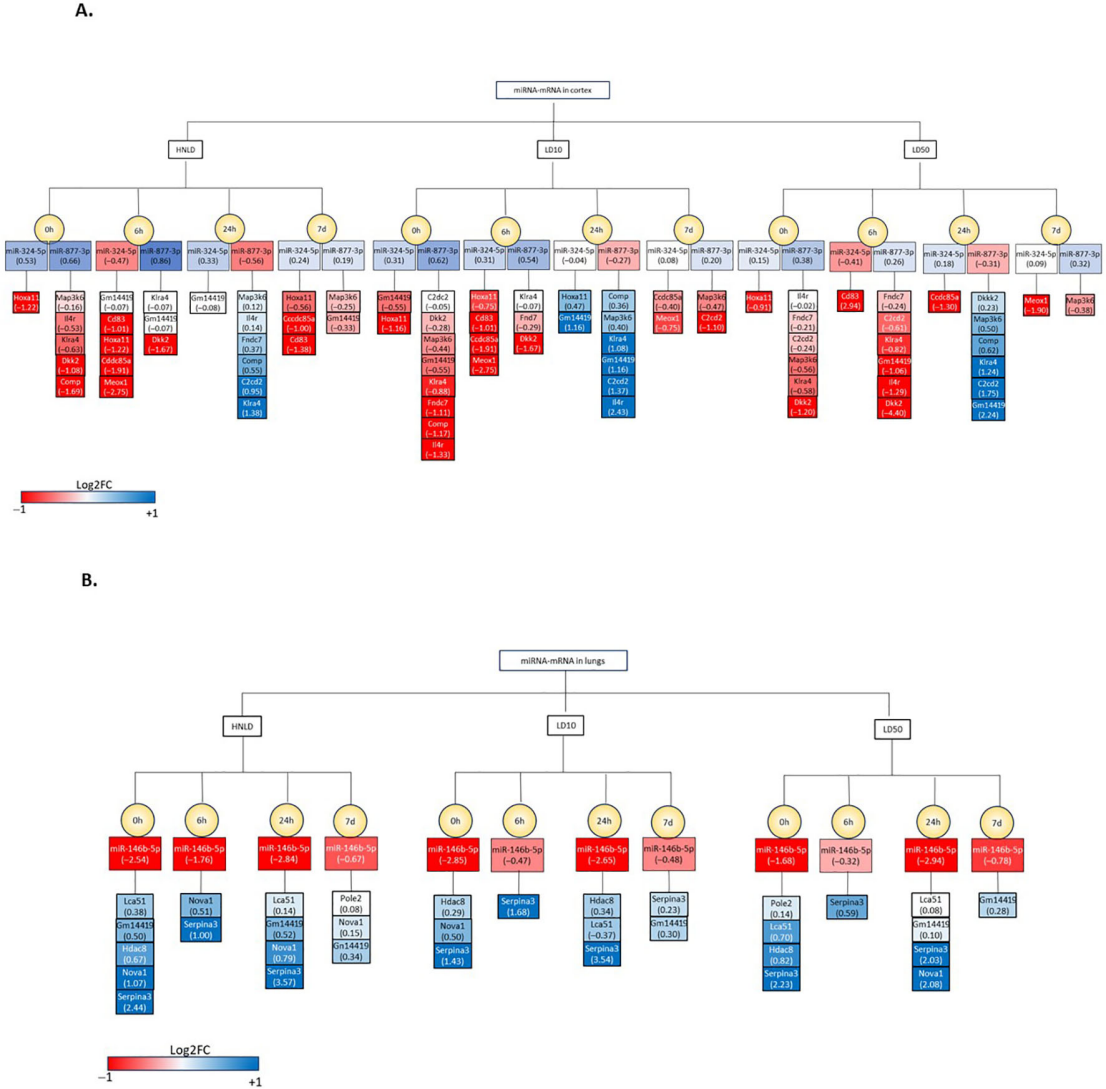
List of the miRNA-mRNA showing largely consistent regulation across the doses and time since exposure. The log_2_(fold change) values are in parenthesis next to the name of miRNAs and mRNAs. The legend of color bar is shown at the bottom of the figure. **(A)** miRNA-mRNA in cortex. Two miRNAs, namely *miR-324-5p* and *miR-877-3p*, and their downstream gene targets are featured. **(B)** miRNA-mRNA in lungs. *miR-146-5p* and its downstream gene targets are featured.

*miRNA-mRNA integration:* **Figure 7A** lists the most consistent miRNA-mRNA axes in the cortex. To note: miR-324-5p-HOXA11, miR-877-3p-DKK, and miR-877-3p-MAP3K6 emerged as the leading cortical miRNA-mRNA axis that showed a consistent regulatory profile across the dose and time gradient. Likewise, **Figure 7B** listed the most consistent miRNA–mRNA axes in the lungs, and miR-146b-5p-SERPINA3 emerged as the leading pulmonary miRNA–mRNA axis that showed a consistent regulatory profile across the dose and time gradient.

## Discussion

The current study builds upon previous findings using the SKH-1 mouse model, which identified the cortex, heart, and lungs as primary targets of fentanyl within the first 24h following LD10 exposure (19). Persistent reductions in glucose uptake were observed in these organs, accompanied by residual activity (19), which is typically triggered by fentanyl-induced rapid hypoxia followed by hyperoxia (7). This hypoxia–hyperoxia cycle eventually arrests brain function and triggers OIRD (32, 33), leading to mortality. Furthermore, the SKH-1 mouse model exhibited atypical cardiac dysregulation resembling WCS (34), a unique comorbidity of fentanyl overdose. Building on this data, the current study investigated the temporal and tissue-specific molecular responses across a time- and dose-gradient to better understand fentanyl’s systemic effects.

Unsupervised PCA plots of mRNA revealed time-dependent impacts of fentanyl in all three organs, suggesting significant transcriptional dysregulation aligned to the past report on PET scan (19). Furthermore, the PCA data highlighted the time-sensitive nature of fentanyl-induced molecular changes, which could be attributed to fentanyl’s rapid tissue distribution due to its high lipophilicity that essentially limits naloxone’s efficacy in reversing overdose (10, 35).

A major finding was the divergent response patterns of these tissues to fentanyl dose gradients. For example, the pulmonary apoptosis-necrosis network was rapidly activated even at the lowest dose, while the heart exhibited dose-dependent latency, with LD50 immediately activating the cardiac apoptosis-necrosis network, whereas HNLD inhibited it at 40 min post-administration. The cortical apoptosis-necrosis network followed a monophasic longitudinal pattern, with early inhibition, activation, and delayed inhibition. In contrast, the cortical immune response showed linear activation over time, co-activating with the pulmonary immune response, suggesting chronic inflammation even at HNLD doses. Persistent cortical immune activation was co-regulated with neurogenic lesion networks, while delayed cardiac apoptosis-necrosis activation was accompanied by pyroptosis, a rapid form of inflammatory cell death (36).

Gene expression analysis revealed tissue-specific molecular changes. In the cortex, genes such as *Tlx2* and *Slc9a2* were downregulated at HNLD and LD10 doses. *Tlx2* is involved in neurodevelopmental regulation (37), and *Slc9a2*, also known as *Nhe2*, is a sodium–hydrogen exchanger and plays a role in ion homeostasis and blood-brain barrier integrity (38). Longitudinal upregulation of cortical genes such as *Mcu* (39), *Cd1d1* (40), *Ccr4* (41), and *Ccr7* (41) highlighted neuronal damage caused by lethal fentanyl doses. In the lungs, inhibited *Sox9* (39) and activated growth-inhibiting *Ltf* at early time points across lethal doses were potentially linked to arrested pulmonary functions (42). Cardiac immune response suppression alongside apoptosis-necrosis network inhibition suggested potential regression of cardiac complications from fentanyl toxicity. Delayed activation of cardiac cell viability and IL-4/IL-13 signaling networks, previously associated with cardiac repair, supported our past proteomics result (18, 19, 43). Consistent upregulation of *Fos* (9) and G-protein coupled receptor (GPCR) signaling networks (44), along with inhibited zinc finger protein-encoding genes (45), indicated decreased signal transmission, a hallmark of fentanyl’s analgesic effects.

The temporal alterations in gene networks were mapped to key phenotypic observations that we have reported in past (18, 19) to better understand their underlying molecular mechanisms. For example, prior to the peak mortality rate following LD50 overdose, networks associated with cell death and immune functions were activated in the heart and lungs but remained inhibited in the cortex. Reduced glucose uptake was observed in all three organs, persisting up to 24h post-exposure. In the brain, reduced glucose uptake during early time points could be attributed to its overall inhibited function, which is particularly detrimental given the brain’s lack of energy reserves and reliance on a continuous glucose supply (32, 33). This observation aligns with the fentanyl-induced biphasic hypoxia-hyperoxia brain response, which is closely connected to OIRD and WCS (7, 32). Notably, the delayed response of the cortical apoptosis-necrosis-immune networks in comparison to that of lungs and heart could be a critical factor in prioritizing the target for early intervention.

Another major finding was the identification of tissue-specific miRNA-mRNA axes, including phylogenetically conserved miRNAs and their downstream gene targets (46). Machine learning analysis (26) identified cortical candidates *miR-324-5p* and *miR-877-3p* and pulmonary candidate *miR-146b-5p*, which have demonstrated phylogenetically conserved and functionally similar characteristics between humans and mice. miR-324-5p and miR-877-3p are linked to neuronal injury and apoptosis, respectively (47, 48), with miR-877-3p being investigated as a therapeutic target for stroke, a pathological endpoint of hypoxia, which is a comorbidity of fentanyl-induced neurotoxicity (7, 49). Cardiac miRNA-mRNA axes included *miR-324-5p–Hoxa11*, *miR-877-3p–Dkk*, and *miR-877-3p–Map3k6*, which regulate cell death, proliferation, and immune responses. Pulmonary *miR-146b-5p* is negatively correlated with enhanced cell migration and invasion (50, 51), while *miR-449a-5p* is associated with cell viability (52). A consistently regulated *miR-146b-5p–Serpina3* axis is linked to immune pathways and tissue injury (53).

### Study limitations

The study acknowledges several limitations inherent to preclinical pharmacology research, particularly in the context of fentanyl exposure. First, the use of a ‘pure’ form of fentanyl differs from “street fentanyl,” which often contains impurities and adulterants. This distinction limits the applicability of the present findings to real-world scenarios of drug abuse (3), where poly-drug use (e.g., alcohol, cocaine, xylazine, methadone) is common. Consequently, the biological consequences of poly-drug use were beyond the scope of current study.

Hypoxia is one of the major and rapid implications of fentanyl (7), and there is a possibility that hypoxia could be followed by a hyperoxic phase in the surviving cohort. Current study was unable to demultiplex the impacts of hypoxia-exclusive molecular implications from the fentanyl related effects. While treating hypoxia in conjunction with fentanyl abuse is a logical strategy, understanding the hypoxia-specific molecular targets could offer unique advantages.

The use of immunocompetent SKH-1 mice minimized stress-induced confounding factors by bypassing pre-imaging hair removal. However, baseline pharmacokinetic data for fentanyl in this model were limited, necessitating a *de-novo* establishment of baseline parameters. Study parameters, including dose-response curves and time durations linked to clinical manifestations, were customized for the SKH-1 mouse model (18, 19).

Other limitations included a small sample density of four mice per study group and a male-biased model. Two-dimensional PCA plots explained less than 30% of the total variance. These limitations were addressed by conducting a multi-omics assay across 64 mice, mapping over 25 million reads of mRNA profiles and 5 million reads of miRNA profiles, spanning a wide time- and dose-range. State-of-the-art analytical tools with strict statistical thresholds were employed, ensuring robust data analysis. This high-density profiling of the molecular landscape likely reduces the necessity for additional validation methods, providing a reliable dataset with high confidence.

The study’s focus on miRNAs addressed several limitations. By screening for miRNAs that are sequentially conserved and functionally similar between humans and mice, the research identified molecular features likely to play fundamental roles in gene regulation, cell signaling, and development. These conserved miRNAs provide insights into shared biological mechanisms across species and offer potential for miRNA-based interventions, such as mimics or inhibitors, with translational relevance to humans.

Finally, the average time of death post-LD50 exposure (~44 min) introduced potential survivor bias, as data collection continued from 6h or later (18, 19). To mitigate this, the study carefully monitored physical and behavioral attributes to determine optimal terminal time points for organ collection. Despite these limitations, the study employed robust methodologies and analytical tools to deliver meaningful insights into fentanyl’s molecular and physiological effects.

## Conclusion

In conclusion, the current study successfully utilized a specific rodent strain to integrate imaging data with omics results, providing a comprehensive analysis of tissue-specific molecular responses to fentanyl overdose. By customizing assay parameters and employing advanced analytical tools, the study identified phylogenetically conserved miRNA–mRNA targets with high translational potential, offering valuable insights for the development of next-generation therapeutic interventions. Furthermore, the identification of variable organ response profiles to a dose gradient highlights the potential for precision drug delivery systems to rapidly mitigate fentanyl toxicity. These findings contribute to a deeper understanding of fentanyl’s molecular effects and pave the way for targeted and effective therapeutic strategies.

## Data Availability

The datasets presented in this study can be found in online repositories. The names of the repository/repositories and accession number(s) can be found here: GSE296051and GSE296604 (GEO).

## Author disclaimer

Material has been reviewed by the Walter Reed Army Institute of Research. There is no objection to its presentation and/or publication. The opinions or assertions contained herein are the private views of the author, and are not to be construed as official, or as reflecting true views of the Department of the Army or the Department of Defense. Research was conducted under an IACUC-approved animal use protocol in an AAALAC International - accredited facility with a Public Health Services Animal Welfare Assurance and in compliance with the Animal Welfare Act and other federal statutes and regulations relating to laboratory animals.

## Supplementary material

Supplementary Figure 1 Significantly altered gene regulation patterns across the time since fentanyl exposure. The regulation profiles are plotted against the time since exposure. Those genes, which showed significant negative slope with time were boxed in the legend with a label “longitudinally downregulated.” Major biofunctions associated with each set of genes are presented in a box. **(A–C)** Cortex, **(A)** Cortex**-**HNLD. **(B)** Cortex-LD10. **(C)** Cortex-LD50. **(D–E)**: Heart. **(D)** Heart-LD10. **(E)** Heart-LD50. Note, no cardiac gene at HNLD met this criteria. **(F–H)**: Lungs. **(F)** Lungs-HNLD. **(G)** Lungs-LD10. **(H)** Lungs-LD50.

Supplementary Figure 2 Biofunction related to cell death. **(A–C)**: The molecular clusters include oval and rectangular nodes, which represent the genes and the sub-networks, respectively. The edges represent the relationships between the nodes. **(A)** Cortex, **(B)** Heart and **(C)** Lungs. **(D-E)** The hierarchical clusters of the genes linked to cell death. **(D)** 56 genes linked to cortical cell death. **(E)** 38 genes linked to cardiac cell death. **(F)** 70 genes linked to pulmonary cell death.

Supplementary Figure 3 Hierarchical clusters of the functional networks enriched by genes differentially expressed by dose × time. The color code is on the right-hand side. **(A–C)**: apoptosis, necrosis, and pertinent networks in **(A)** Cortex, **(B)** Heart, **(C)** Lungs. **(D–F)**: Immune response networks. **(D)** Cortex, **(E)** Heart, and **(F)** Lungs.

Supplementary Figure 4 Longitudinal profile of the z-scores of multi-tissue networks. The X-axis represents the time since fentanyl exposure and Y-axis represents the z-scores of the networks; z-score ≥ 1.0: activated network and z-score ≤ −1.0: inhibited network. **(A–C)**: Cortical cell death networks and its correlated networks across the tissues **(A)** Cortex- HNLD, **(B)** Cortex- LD10, **(C)** Cortex-LD50. **(D–F)**: Cardiac cell death networks and its correlated networks across the tissues **(D)** Heart- HNLD, **(E)** Heart- LD10, **(F)** Heart-LD50. **(G–I)**: Pulmonary cell death networks and its correlated networks across the tissues **(A)** Lungs- HNLD, **(B)** Lungs- LD10, **(C)** Lungs-LD50.

Supplementary Figure 5 The Venn diagram shows the number of miRNAs, which are sequentially and functionally conserved between humans and mice. The conserved miRNAs between the two arms of **Figure 1B** are labeled as features with high translational potential. **(A)** Cortex, **(B)** Heart, **(C)** Lungs.

Supplementary Figure 6 Differentially expressed cortical miRNAs across the dose and time since the exposure identified by a series of Venn analysis. A hierarchal cluster of the 6 most conserved miRNAs is shown.

Supplementary Figure 7 Differentially expressed pulmonary miRNAs across the dose and time since the exposure identified by a series of Venn analysis. A hierarchal cluster of the 19 most conserved miRNAs is shown.

Supplementary Table 1 Functional networks enriched by differentially expressed mRNA, which were cumulatively regulated by dose and time since fentanyl administration.

Supplementary Table 2 List of miRNAs generated by the ML-algorithm towards curating the conserved candidates between human and mice.

Supplementary Table 3 List of miRNAs and their downstream mRNA targets.
