# Supplementary figures and images for: Fentanyl-induced cortical and cardiopulmonary damage linked to immune response functions and apoptosis-necrosis networks in a multi-omics mouse model
Source: Front Immunol. 2026 Mar 23;17:1694651. doi: 10.3389/fimmu.2026.1694651 (PMC13051511; doi:10.3389/fimmu.2026.1694651)

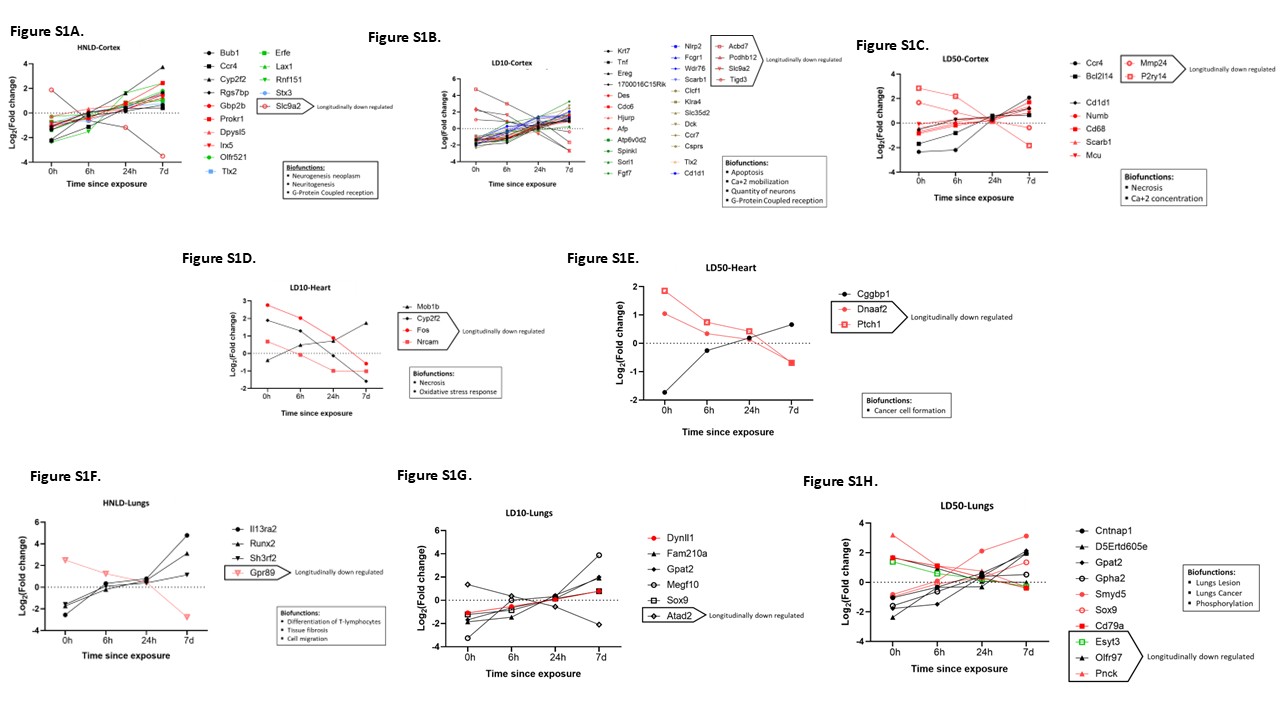

Supplement: Supplementary Figure 1 — Significantly altered gene regulation patterns across the time since fentanyl exposure. The regulation profiles are plotted against the time since exposure. Those genes, which showed significant negative slope with time were boxed in the legend with a label “longitudinally downregulated.” Major biofunctions associated with each set of genes are presented in a box. (A–C) Cortex, (A) Cortex-HNLD. (B) Cortex-LD10. (C) Cortex-LD50. (D–E): Heart. (D) Heart-LD10. (E) Heart-LD50. Note, no cardiac gene at HNLD met this criteria. (F–H): Lungs. (F) Lungs-HNLD. (G) Lungs-LD10. (H) Lungs-LD50. [file Image1.jpeg]

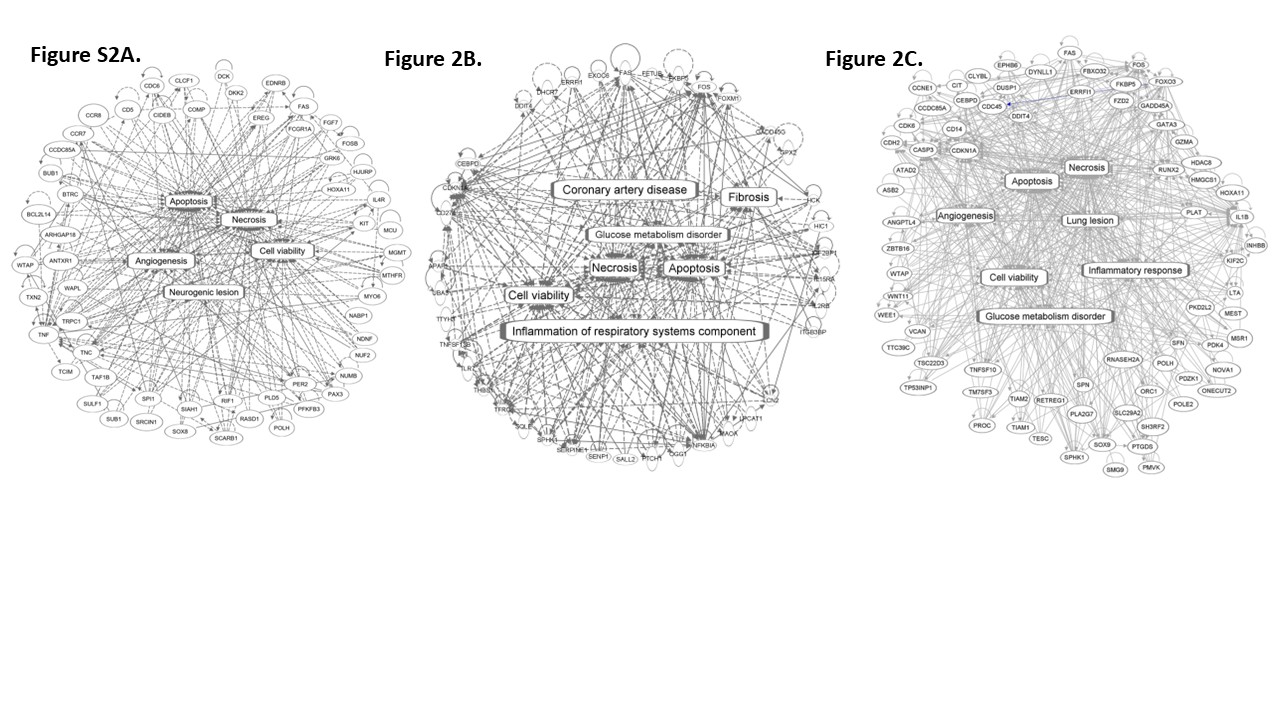

Supplement: Supplementary Figure 2 — Biofunction related to cell death. (A–C): The molecular clusters include oval and rectangular nodes, which represent the genes and the sub-networks, respectively. The edges represent the relationships between the nodes. (A) Cortex, (B) Heart and (C) Lungs. (D-E) The hierarchical clusters of the genes linked to cell death. (D) 56 genes linked to cortical cell death. (E) 38 genes linked to cardiac cell death. (F) 70 genes linked to pulmonary cell death. [file Image2.jpeg]

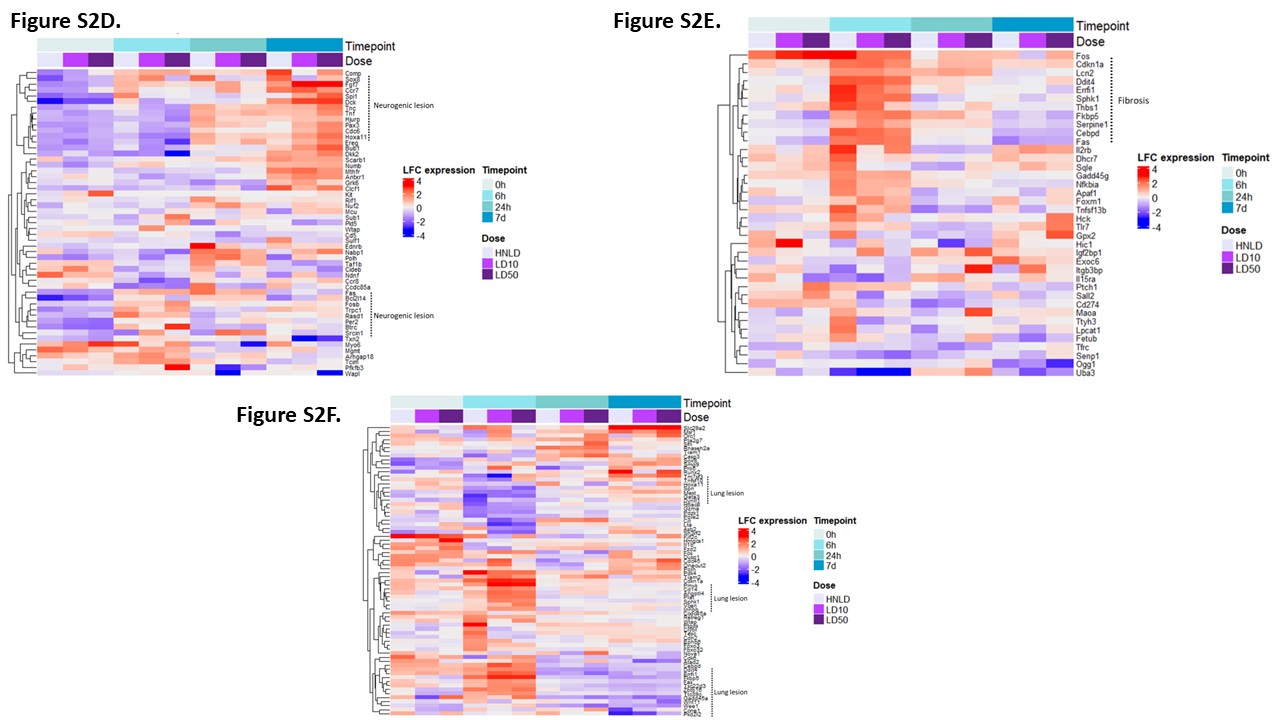

Supplement: Supplementary Figure 3 — Hierarchical clusters of the functional networks enriched by genes differentially expressed by dose × time. The color code is on the right-hand side. (A–C): apoptosis, necrosis, and pertinent networks in (A) Cortex, (B) Heart, (C) Lungs. (D–F): Immune response networks. (D) Cortex, (E) Heart, and (F) Lungs. [file Image3.jpeg]

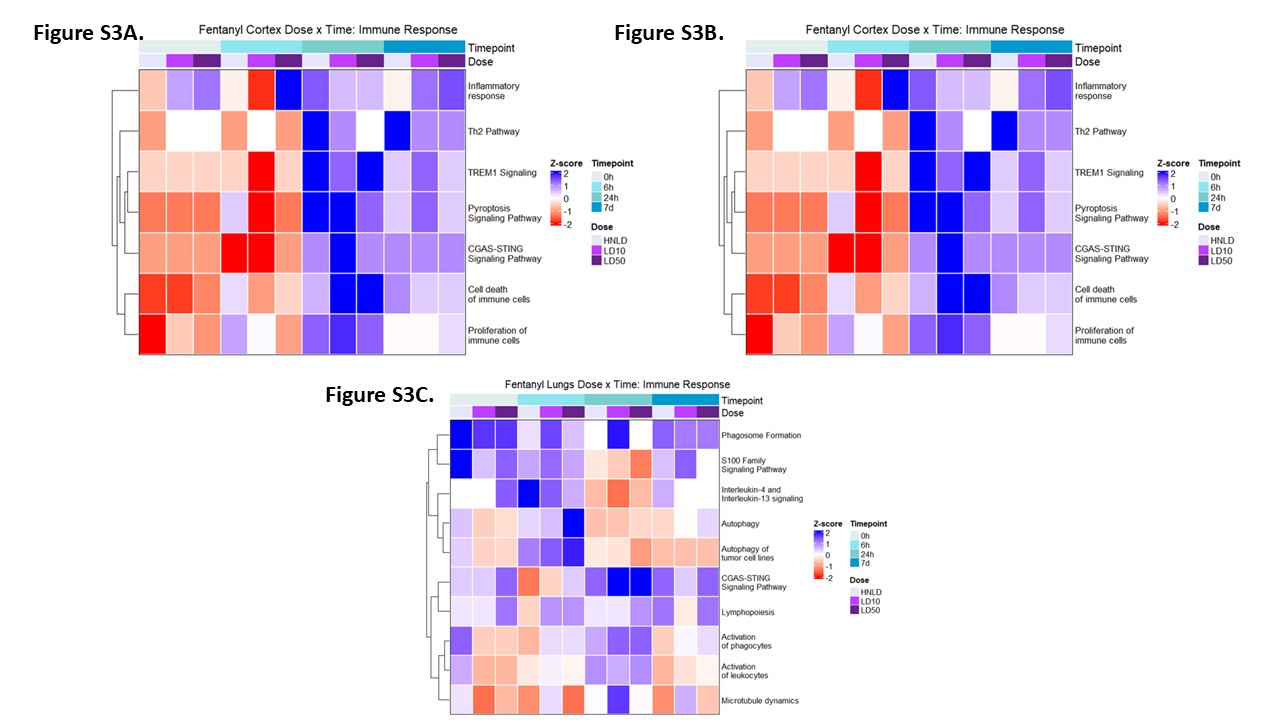

Supplement: Supplementary Figure 4 — Longitudinal profile of the z-scores of multi-tissue networks. The X-axis represents the time since fentanyl exposure and Y-axis represents the z-scores of the networks; z-score ≥ 1.0: activated network and z-score ≤ −1.0: inhibited network. (A–C): Cortical cell death networks and its correlated networks across the tissues (A) Cortex- HNLD, (B) Cortex- LD10, (C) Cortex-LD50. (D–F): Cardiac cell death networks and its correlated networks across the tissues (D) Heart- HNLD, (E) Heart- LD10, (F) Heart-LD50. (G–I): Pulmonary cell death networks and its correlated networks across the tissues (A) Lungs- HNLD, (B) Lungs- LD10, (C) Lungs-LD50. [file Image4.jpeg]

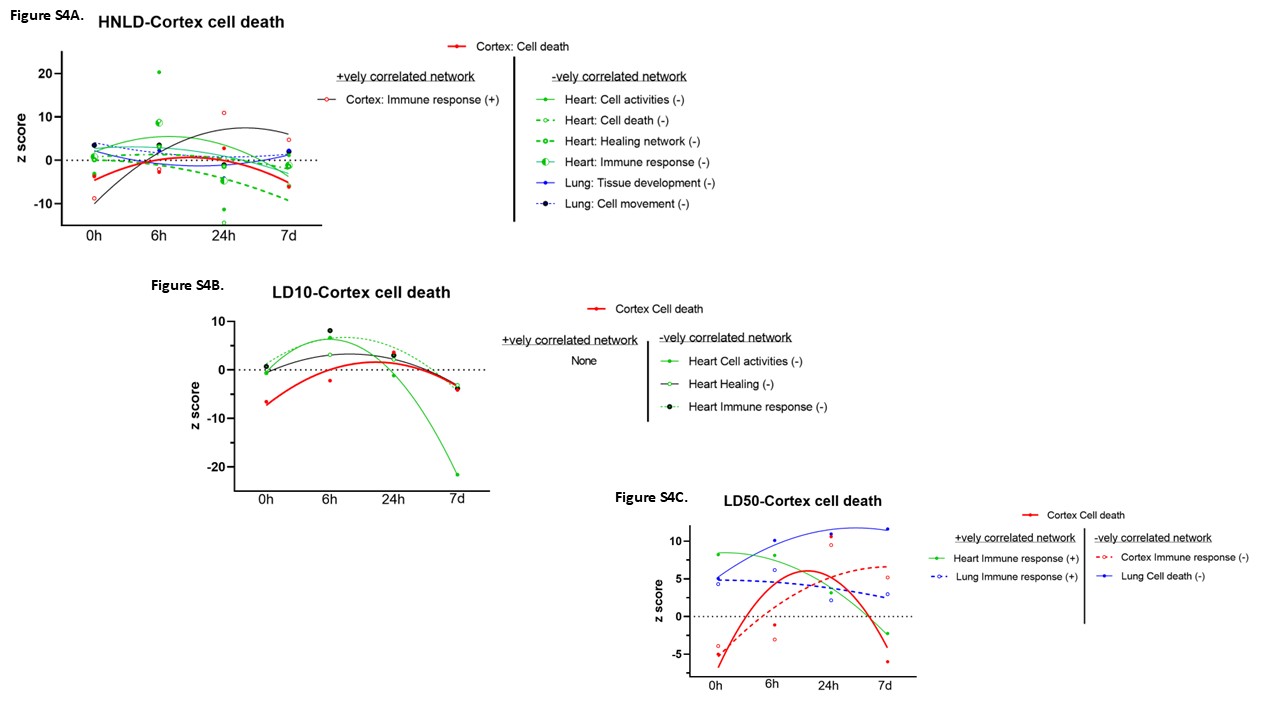

Supplement: Supplementary Figure 5 — The Venn diagram shows the number of miRNAs, which are sequentially and functionally conserved between humans and mice. The conserved miRNAs between the two arms of Figure 1B are labeled as features with high translational potential. (A) Cortex, (B) Heart, (C) Lungs. [file Image5.jpeg]

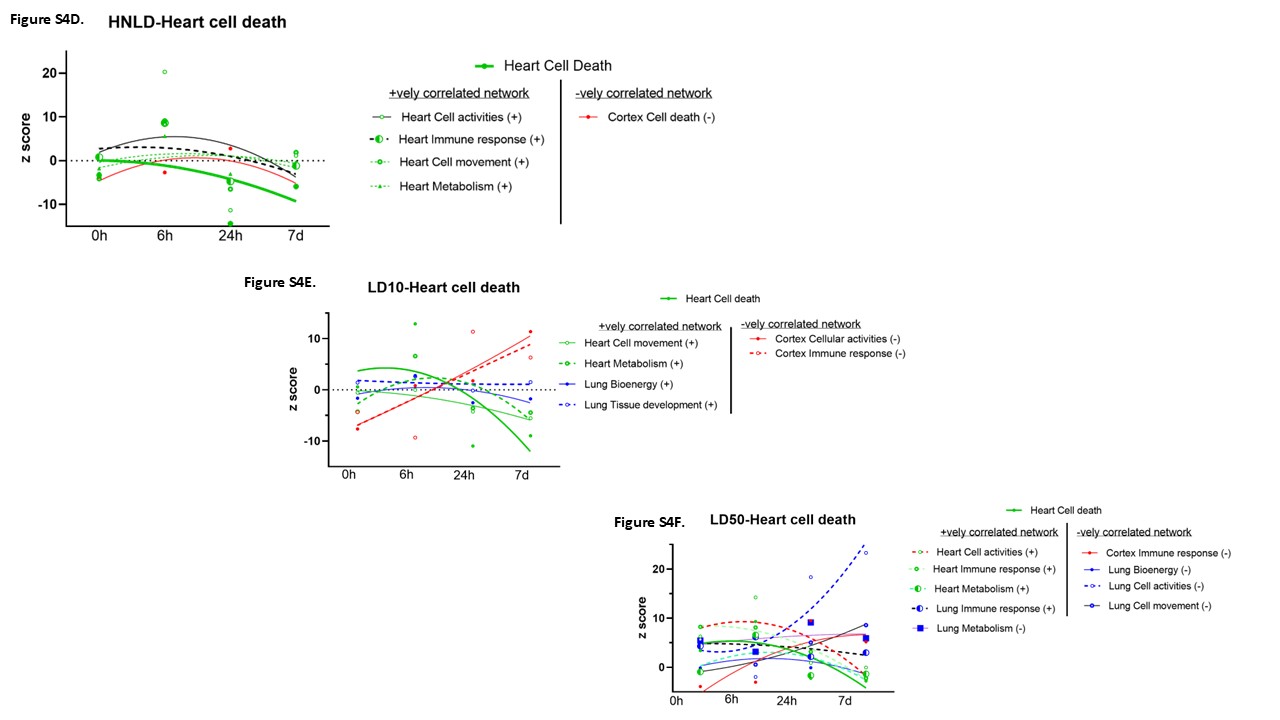

Supplement: Supplementary Figure 6 — Differentially expressed cortical miRNAs across the dose and time since the exposure identified by a series of Venn analysis. A hierarchal cluster of the 6 most conserved miRNAs is shown. [file Image6.jpeg]

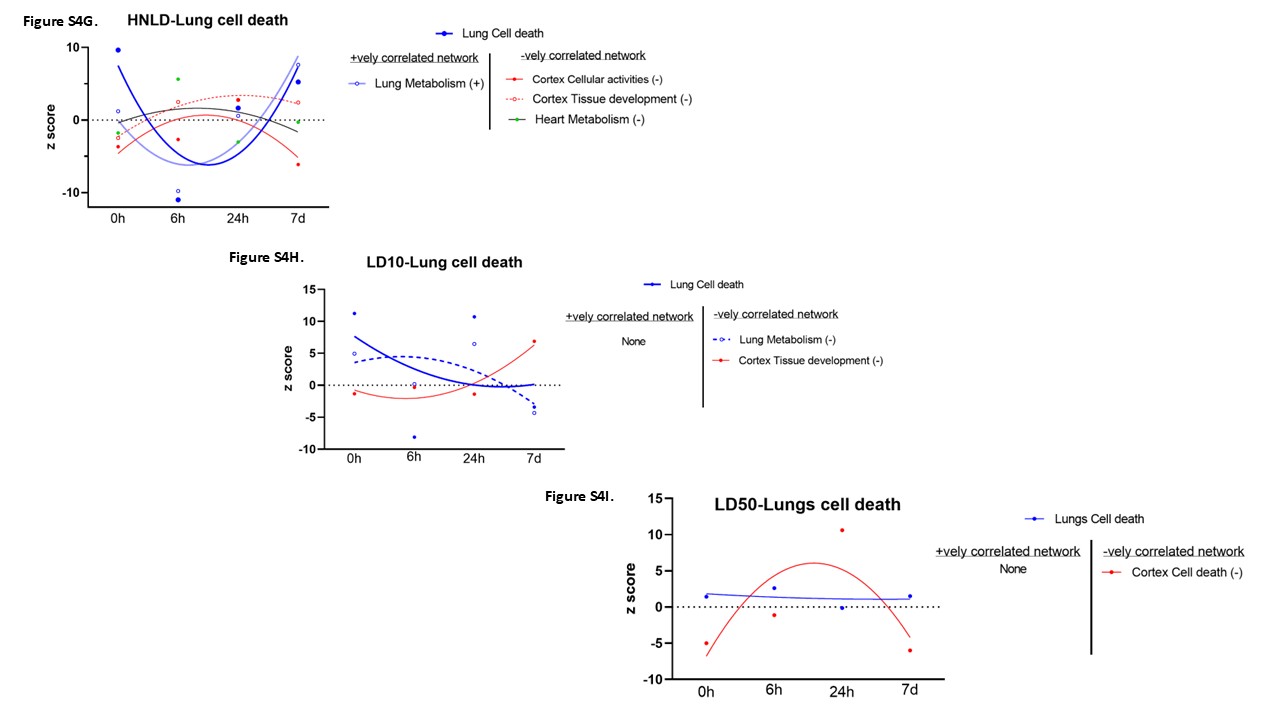

Supplement: Supplementary Figure 7 — Differentially expressed pulmonary miRNAs across the dose and time since the exposure identified by a series of Venn analysis. A hierarchal cluster of the 19 most conserved miRNAs is shown. [file Image7.jpeg]

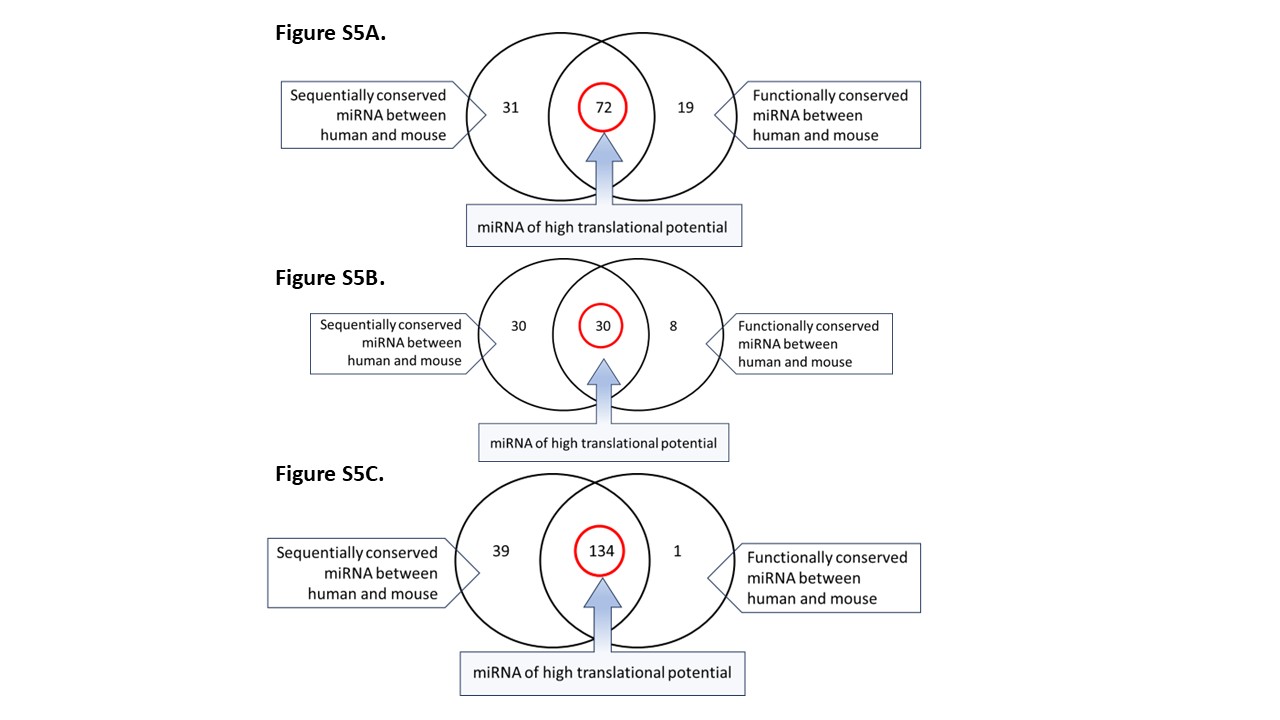

Supplement: Supplementary file 8 [file Image8.jpeg]

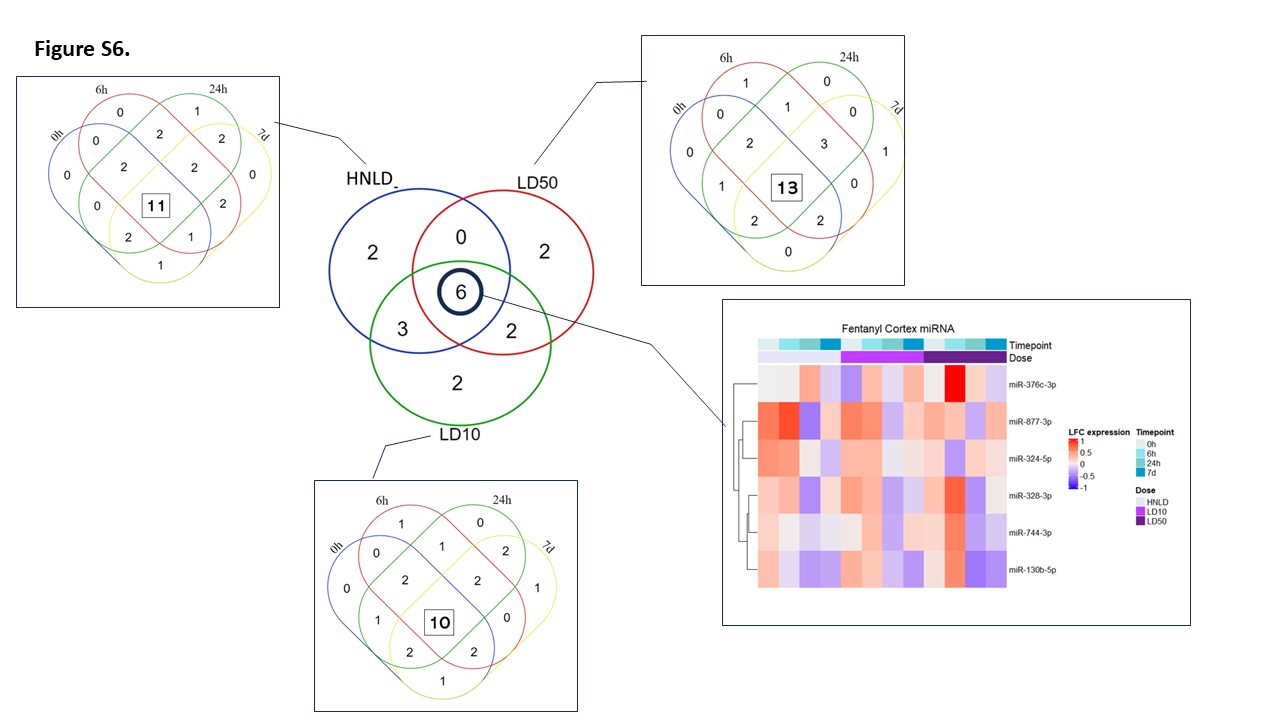

Supplement: Supplementary file 9 [file Image9.jpeg]

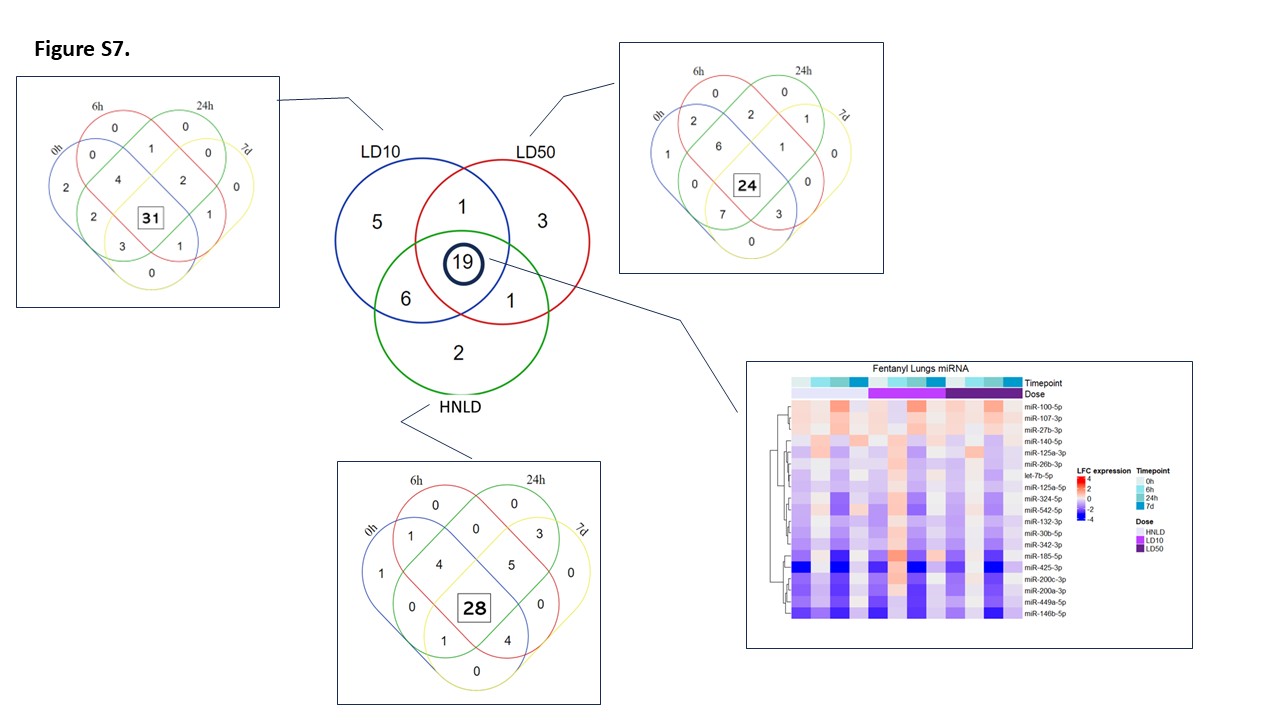

Supplement: Supplementary file 10 [file Image10.jpeg]
